# Possible Mode of Spread of Hepatitis B and Hepatitis C in Chronic Liver Disease Patients Presenting at CMH Lahore Medical College and Institute of Dentistry

**DOI:** 10.7759/cureus.6863

**Published:** 2020-02-03

**Authors:** Faiqua Yasser, Rubab Sherazi, Raja Y Shahbaz, Nedal Iqbal, Sadia Iqbal, Rizwana Kamran

**Affiliations:** 1 Oral Pathology, Institute of Dentistry, CMH Lahore Medical and Dental College, National University of Medical Sciences, Lahore, PAK; 2 Operative Dentistry, Institute of Dentistry, CMH Lahore Medical and Dental College, National University of Medical Sciences, Lahore, PAK; 3 Gastroenterology, Hameed Latif Hospital, Lahore, PAK; 4 Oral Biology, Fatima Memorial Hospital, Lahore, PAK; 5 Oral Pathology, Lahore Medical and Dental College, Lahore, PAK; 6 Medical Education and Simulation, CMH Lahore Medical and Dental College, National University of Medical Sciences, Lahore, PAK

**Keywords:** hepatitis b and c, chronic liver disease, cirrhosis, hepatocellular carcin

## Abstract

Introduction

Chronic hepatitis B and C are the leading causes of chronic liver disease and a significant cause of mortality and morbidity worldwide. Hepatitis B is a preventable disease with vaccination, which is available worldwide. About 257 million people are affected worldwide with hepatitis B and around 71 million people with hepatitis C, and Pakistan is the second most prevalent country with hepatitis C. The possible mode of spread of hepatitis B and C in chronic liver disease patients presenting at CMH medical wards and dental clinics was evaluated. Among various types of hepatitis, A, B, and C are the most common. The course of disease followed by hepatitis A is short term, but when we talk about the common types, which are B and C, the disease is chronic and, moreover, the complications associated with these types are more severe too.

Materials and methods

The study was carried out at the medical wards and dental clinics of CMH Lahore Medical College. A total of 240 patients were evaluated for the possible mode of spread of hepatitis B and C. A detailed history regarding the use of needles, surgical procedures, including dental treatment, unsterilized razors for shaving (barbers'), blood transfusions, tattooing, and mode of delivery in females, was evaluated and data were interpreted.

Aims and objectives

To observe the mode of spread of hepatitis B and C among patients of CMH Lahore Medical College and Institute of Dentistry.

Results

The results of our study revealed that the most common mode of spread of disease in males was exposure to infected blades and instruments at barbers' shops. In females, blood transfusion and caesarian section, especially in rural areas, remained the most common possible cause of spread. A small number of patients was not aware of the possible mode of transmission, whereas a few others did not seek treatment for the disease.

Conclusion

From our study, we can conclude that a substantial number of patients with chronic liver disease have exposure to various risk factors. Exposure to the unsterilized equipment of barbers and improperly screened blood remains the main cause of the spread of hepatitis B and C in males whereas surgical procedures related to gynecological procedures and blood transfusions remain the second most common cause. Dental procedures in the hands of trained dental professionals/dentists carry less danger of transmitting the disease but carry an equally high incidence if quacks do the dental procedures.

## Introduction

Chronic hepatitis B and C are inflammatory conditions of the liver and are caused by hepatitis B and C viruses worldwide eventually leading to cirrhosis of the liver and hepatocellular carcinoma [1]. Hepatitis B is a preventable disease and vaccination can be done to prevent the disease, whereas hepatitis C cannot be prevented with vaccination [2]. Mode of spread of hepatitis B is infected bodily fluids and hepatitis C infection is blood-borne [3].

According to the World Health Organization (WHO), nearly 257 million people are affected with hepatitis B worldwide and a disease burden of 2% in South East Asia. Globally, 71 million people have hepatitis C and Pakistan is the second most affected country with as high as 5% of the population suffering from chronic hepatitis C [4].

Hepatitis B usually spreads through unsafe sexual practice, infected needles, vertical transmission from mother to fetus, surgical and dental procedures, and blood transfusions [5-7].

Hepatitis C is also transmitted through blood transfusions, intravenous drug abusers, use of unsterilized razors for shaving (barbers), nose piercing, and infected needles or instruments during surgical procedures [8-9].

Many people who are suffering from this chronic infection of hepatitis B and C are not aware of this disease until it becomes chronic and advanced and complications are there due to the end stage of the disease, which is liver cirrhosis.

The symptoms of the acute and chronic stages of hepatitis almost remain similar for B and C types. Therefore, it is difficult to distinguish between acute and chronic stages clinically. Moreover, it is not possible to identify on a clinical basis, from which the type of hepatitis is the patient suffering until a proper history and lab diagnosis is available. Patients can present with flu-like symptoms, such as malaise, headache, joint pains, lethargy, dark yellow urine, pale or grey stools, jaundice, nausea, vomiting, or persistent low-grade fever. According to the literature, about 15%-25% people develop chronic liver disease. Chronic liver disease includes damage to the liver, cirrhosis of the liver, and the most severe complication is hepatocellular carcinoma. Some people completely elude clinical signs and symptoms of the disease until found out through screening done for other disorders.

## Materials and methods

This cross-sectional study was carried out at the medical wards of CMH Lahore Medical College Institute of Dentistry from January 2017 to September 2018, over a period of one year and nine months. A total number of 240 patients were admitted with chronic liver disease. Both male and female patients were included in the study with the age group of 18-60 years. Written consent was taken from all patients and a proforma containing a brief history and vaccination record for hepatitis B was filled along with the possible risk factor and its exposure.

The major risk factors evaluated during the study were the use of needles, blood transfusions, mode of delivery in females, any surgical procedure, any dental procedure in life, tattooing, and frequent visits to barbers with the use of blades for shaving and haircuts.

Aims and objectives included information regarding the prevalence of hepatitis B and C according to age and gender, as well as, to observe the possible mode of spread.

Data were analyzed using IBM SPSS Statistics version 22.0 (IBM Corp., Armonk, NY).

## Results

Out of a total of 240 patients, 160 (66%) were males and 80 (34%) were females. One-hundred seventy-six (73%) patients were found to be hepatitis C positive while 64 (23%) patients were hepatitis B positive. Out of 80 females, 60 were hepatitis C positive and 20 were hepatitis B positive. Similarly, out of 160 male patients, 44 were hepatitis B positive and the remaining 116 were hepatitis C positive. According to the division of age, we made two groups, 18 to 30 and 30 to 60. The prevalence of disease according to different age groups is shown in Table 1.

**Table 1:** Distribution of hepatitis B and C according to age

AGE GROUPS	HEPATITIS B	HEPATITIS C	TOTAL
18 – 30 YEARS	42 (24 %)	132 (76 %)	174
31 – 60 YEARS	22 (33 %)	44 (67 %)	66

The prevalence of hepatitis B and C was higher in the age group of 18 to 30 years. Similarly, the prevalence of hepatitis B was more in males and less in females and hepatitis C was more prevalent in females, as shown in Table 2.

**Table 2:** Gender-wise distribution of hepatitis B and C patients

GENDER	HEPATITIS B	HEPATITIS C	TOTAL
Male	44 (27.5 %)	116 (72.5 %)	160
Female	20 (25 %)	60 (75 %)	80

Of the potential risk factors, availing the facilities of barbers was the most frequent in males for the spread of infection, as out of 160 males, 110 (68.75%) were associated with this risk factor followed by blood transfusions in 20 (12.5 %), surgical procedures in 10 (6.25%), whereas in the remaining 20 patients, no risk factor could be identified. In females, blood transfusions and cesarean sections remained the most prevalent risk factors - 35 (43.5%) and surgical 20 (25%), respectively, the remaining 25 patients remained undiagnosed for the route of acquiring hepatitis B and C.

## Discussion

Hepatitis B and C are very common among patients presenting at tertiary care hospitals for the treatment of various diseases. Patients should be made aware of the fact that they are carrying the disease and should seek proper treatment. Awareness regarding further prevention of the disease and evaluating the patients for the possible cause of the spread of disease is important. Awareness regarding the possible mode of spread reduces the risk of further re-infection and gives suggestions to patients on how to prevent disease in their social setup.

This study is consistent with other studies at different centers. A similar study conducted at the Armed Forces Institute of Dentistry (AFID) shows a prevalence of 4.6% of hepatitis b and c in the screened population [10]. Similarly, a study done at Ayub Medical College shows a prevalence of 4.2% in the screened population [11].

From our study, it can be seen that barbers remained the main source of the spread of the virus among the male population followed by blood transfusions and surgical procedures. Similarly in female’s blood transfusions and surgical procedures, such a cesarean section, remain a high cause of the spread of disease among females.

Patients were made aware that getting blood screened prior to transfusion and ensuring adequate sterilization of instruments are essential for safe surgical practice. Males were advised to give their own shaving equipment to barbers to reduce the risk of acquiring the disease.

## Conclusions

From our study, we can conclude that a substantial number of patients with chronic liver disease have exposure to various risk factors. Exposure to the unsterilized equipment of barbers and improperly screened blood remains the main cause of the spread of hepatitis B and C in males whereas surgical procedures related to gynecological procedures and blood transfusions remain the second most common cause.
